# Genistein protects against Aβ_25–35_ induced apoptosis of PC12 cells through JNK signaling and modulation of Bcl-2 family messengers

**DOI:** 10.1186/s12868-016-0329-9

**Published:** 2017-01-12

**Authors:** Fuling You, Qiao Li, Guifang Jin, Yaojie Zheng, Jingrong Chen, Hong Yang

**Affiliations:** Basic Medical College, Guangdong Pharmaceutical University, Guangzhou, 510006 Guangdong China

**Keywords:** Alzheimer’s disease, Aβ_25–35_, Apoptosis, Genistein, JNK, Bcl-2

## Abstract

**Background:**

Deposition of aggregated amyloid beta (Aβ) protein is hallmark of Alzheimer’s disease, leading to dysfunction and apoptosis of neurons. The isoflavone phytoestrogen compound genistein (Gen) exerts a significant protective effect against Aβ_25–35_ induced neurotoxicity and mitochondrial damage in rat pheochromocytoma (PC12) cells. However, the mechanisms underlying Gen’s rescue remain elusive. Therefore we endeavored to research further the molecular mechanisms underlying Gen’s inhibition of Aβ_25–35_ induced apoptosis of neurons.

**Results:**

We found that Gen dramatically suppressed the activation by Aβ_25–35_ of p-c-Jun N-terminal kinase (p-JNK), and also inhibited the JNK-dependent decreased of Bcl-w and increased of Bim. Furthermore, Gen significantly reduced the cytoplasmic concentrations of cytochrome c and Smac protein as well as caspase-3 activity. Additionally, pretreatment with JNK inhibitor SP600125 effectively suppressed Aβ_25–35_ induced PC12 cell cytotoxicity.

**Conclusion:**

Taken together, the results suggested that Gen protects PC12 cells from Aβ_25–35_ induced neurotoxicity by interfering with p-JNK activation, thus attenuating the JNK-dependent apoptosis through the mitochondrial pathway. These findings constitute novel insights into the pathway for Aβ_25–35_ toxicity, and the neuroprotective action of Gen.

**Electronic supplementary material:**

The online version of this article (doi:10.1186/s12868-016-0329-9) contains supplementary material, which is available to authorized users.

## Background

The amyloid β protein is a β-sheet peptide compose by 39–43 amino acid residues; its fragment Aβ_25–35_ (GSNKGAIIGLM), the smallest fragment formed by brain proteases, retains the toxicity of the parent peptide as well as a capacity to form aggregates in vivo. As such, Aβ_25–35_ promotes to the development of Alzheimer’s disease (AD) pathology and the resultant clinical symptoms [1, 2]. Deposition of Aβ_25–35_ in brain triggers tau protein phosphorylation and formation of intracellular neurofibrillary tangles (NFT), subsequently leading to mitochondrial dysfunction and membrane rupture, which then proceeds to necrosis or apoptosis [3]. As such, by investigating the pathway by which Aβ_25–35_ toxicity leads to neuronal apoptosis we may understand the development of new treatment strategies in AD.

Extensive research has shown that Aβ_25–35_ accumulation is linked with many signaling pathways implicated in neurodegenerative disease. In particular, c-Jun N-terminal kinase (JNK) is a factor in Aβ induced apoptosis of neurons [4]. JNK regulates many transcription factors, including the Bcl-2 family [5], which importantly controls of the mitochondria apoptosis pathway [6]. Finally, the release of the mitochondrial proteins cytochrome c and second mitochondrion-derived activator of caspase (Smac) into cytoplasm is an important indicator of cellular apoptosis [7].

Genistein (Gen) is an isoflavone phytoestrogen derived from soybeans. It is present in tofu (soybean curd) and its consumption has shown promising results as a moderator of cognitive deficits in AD [8]. Indeed, substantial evidence suggests that Gen inhibits Aβ_25–35_-induced toxicity via regulation of many relevant signaling pathways [9–11] and by facilitation of Aβ clearance from the nervous system [12]. Conversely, another study showed that dietary Gen can lead to cognitive impairment [13]. Resolving these contradictory findings requires a better understanding of the molecular mechanisms whereby Gen modulates AD neuropathology. However, differentiated neuron-like rat pheochromocytoma (PC12) cells often used for studying neuroprotection [14, 15].

Given this background, we endeavored to use PC12 cells in order to test whether Gen protects PC12 cells from Aβ_25–35_ induced neurotoxicity. In particular, we researched the regulatory effects of Gen on the expression of Bcl-2 family members (such as the Bcl-w and Bim) in PC12 cells challenged with Aβ_25–35_ in vitro. Furthermore, we also examined the effects of Gen on the JNK phosphorylation level upstream in the mitochondrial apoptotic pathway. Our studies in PC12 cells address fundamental aspects of the potential of Gen to ameliorate Aβ-induced neuronal cytotoxicity and apoptotic signaling.

## Methods

### Cell culture

PC12 cells were obtained from the Institute of Biochemistry and Cell Biology (Shanghai, China) cell bank and cultured in Roswell Park Memorial Institute (RPMI) 1640 medium supplemented with 10% v/v fetal bovine serum (FBS), 100 U/mL penicillin, and 0.1 mg/mL streptomycin (all from Hyclone, Logan, UT) at 37 °C in a humidified atmosphere of CO_2_ (5%). After every second day, replaced with fresh media, to promote growth until the cells reach a confluence of 70–80%.

### Determination of cell viability

MTT was used to detect viability of PC12 cells. In brief, the cells were cultured in medium at a density 5 × 10^4^ cells per well for 24 h in 96-well plates and then pretreated with or without Gen at final concentrations of 12.5, 25, 50, and 100 μM for 2 h and incubated with Aβ_25–35_ (20 μM) for another 24 h. The Aβ_25–35_ (Sigma-Aldrich) was first dissolved in tri-distilled water at a concentration of 1 mM, and then aged for 3 days in a humidified chamber at 37 °C before being added to the culture medium to the final desired concentration. Cells were added to 10 μL of MTT solution medium (5 mg/mL) and then incubated at 37 °C for 4 h. The medium was then carefully removed, and added 150 μL per well DMSO to dissolve the formazan crystals formed in situ. Cell viability was then determined by measuring the absorbance of each well at 570 nm using a microtiter plate reader (Biotek, VT). Each concentration was repeated three times with five replicates per experiment.

### Hoechst 33342 staining to detect cell apoptosis

Hoechst 33342 (Beyotime Biotechnology, Haimen, China) was used to identify the apoptotic cells. PC12 cells were cultured and then pretreated with Gen at concentrations of 12.5, 25, 50, and 100 μM for 2 h and incubated with Aβ_25–35_ (20 μM) for another 24 h. Cells were washed with PBS and then stained with Hoechst 33342 DNA-binding dye (10 mg/L) for 15 min at 37 °C in darkness. Finally, the cells were washed with PBS and examined under a fluorescence microscope (Leica, Germany).

### Fluorescence-activated cell sorting (FACS) analysis

PC12 cells were cultured in 6-well plates; treated with 12.5, 25, 50, and 100 μM of Gen for 2 h; and finally incubated with Aβ_25–35_ (20 μM) for another 24 h. Cell apoptosis was measured using the Annexin V-FITC Apoptosis Detection Kit I (Beyotime Biotechnology, Haimen, China). In brief, the cells were washed once with PBS and digested with trypsin. The subsequently collected cells were washed once with PBS. Next, 5 μL of Annexin V-FITC and 10 μL of propidium iodide (PI) were then added according to the manufacturer’s instructions. And mixing and incubation for 10 min in darkness at room temperature, cells were detected using a FACS (BD Biosciences, San Jose, CA).

### RNA extraction and real-time RT-PCR quantitation

PC12 cells were treated with Gen at concentrations of 25 μM for 2 h and with the JNK phosphorylation inhibitor SP600125 (Beyotime Biotechnology, Haimen, China) at a concentration of 100 nM for 1 h; finally incubated with Aβ_25–35_ (20 μM) for another 24 h. Total RNA was then isolated from the PC12 cells with Trizol reagent (Invitrogen, Carlsbad, CA). RNA concentration and purity were determined using a fluorospectrophotometer (RF-5301PC; Shimadzu, Japan), and RNA integrity was verified by 1% agarose gel electrophoresis. The first strand cDNAs were synthesized from 2 μg of total RNA in a 20 μL reaction volume using reverse transcriptase (Takara Biotechnology, Dalian, China).

Next, 2 μL portions of the reverse transcription product was amplified with the SYBR^®^ Premix Ex Taq™ II (Tli RNaseH Plus) (Takara Biotechnology, Dalian, China). The special primers were designed from their GenBank sequences and synthesized by Bio Basic Inc. (Shanghai, China): 5′-CACTTTCTACAATGAGCTGCG-3′, 5′-CTGGATGGCTACGTACATGG-3′ for β-actin; 5′-GAGTTTGAGACCCGCTTCC-3′, 5′-GTCCTCACTGATGCCCAGTT-3′ for Bcl-w; and 5′-CTTACACGAGGAGGGCGTTT-3′, 5′-CAGTGCCTTCTCCAGACCAG-3′ for Bim. The thermal profile reactions were performed in a real-time PCR system (Bio-Rad, Hercules, CA), and the amplified products were quantified by measuring the calculated cycle thresholds (CT) for individual targets and the β-actin reference mRNA. The 2^−ΔΔCT^ method was used for quantification and statistical analysis.

### Western blots

Cytoplasmic proteins were isolated using a Cytoplasm Protein Extraction Kit. Thereafter, a BCA Protein Assay Kit (Beyotime Biotechnology, Haimen, China) was used to determine the protein concentrations. The samples were boiled for 5 min. Next, portions containing 20 μg protein were separated on 12% SDS–polyacrylamide gel and transferred onto PVDF membranes (Millipore, Bedford, MA) at a current of 200 mA for 40 min. After blocking for 2 h in a TBS containing 0.1% Tween 20 (TBST) and 5% w/v skim milk powder at room temperature, these membranes were incubated overnight at 4 °C with primary antibodies against β-actin, cytochrome c, and Smac (1:300, BOSTER, Wuhan, China), and anti-p-JNK (1:2000, Cell Signaling Technology, Danvers, MA), with dilutions in TBST. After washing three times with TBST, the membranes were incubated with horseradish peroxidase-conjugated secondary antibody (1:5000, BOSTER, Wuhan, China) for 1 h at room temperature and again washed three or four times. The bands were developed using an ECL kit following the manufacturer’s instructions, with β-actin serving as a loading control. The X-OMAT BT films (Carestream, Xiamen, China) were scanned and quantitated using Quantity One software.

### Caspase activity assay

To evaluate the activity of caspase-3, cell lysates were prepared after their various respective treatments. Assays were performed on 96-well plates by incubating 10 μL portions of cell lysate per sample in 80 μL reaction buffer [0.1% Nonidet P 40, 20 mM Tris-HCl (pH 7.5), 137 mM NAD, and 10% glycerol] containing 10 μL caspase-3 substrate (2 mM, Ac-DEVD-pNA) following the manufacturer’s instructions. Lysates were incubated in this medium at 37 °C for 2 h, and absorbance measured at 405 nm with the microtiter plate reader (Biotek, VT).

### Statistical analyses

Data were expressed as mean ± SD, and all determinations were repeated three times. The data were analyzed by using SPSS v20.0 software (SPSS Inc., Chicago, IL), and *p* < 0.05 were considered statistically significant.

## Results

### Effect of Gen on viability of PC12 cells

**Fig. 1:**
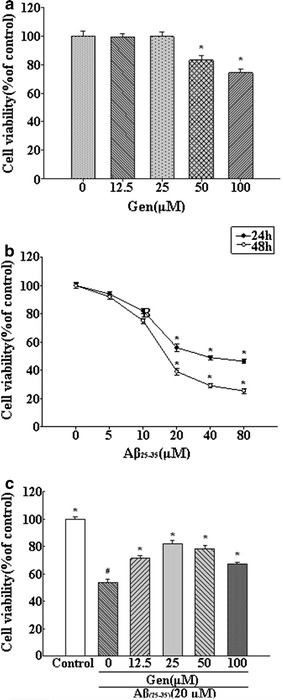
Effect of Gen on the viability of PC12 cell. **a** Viability of PC12 cell treatment with Gen (0, 12.5, 25, 50, and 100 μM) for 24 h. *p < 0.05 compared to control. **b** Dose-dependent change of cell viability of PC12 cells by Aβ treatment for 24 h and 48 h. *p < 0.05 compared to control. **c** Prevention of Aβ_25–35_-induced cell death by Gen. Cells were pretreated with Gen for 2 h followed by exposure to 20 μM Aβ_25–35_ for 24 h. ^#^p < 0.05 compared to control; *p < 0.05 compared to model group. Cell viability was evaluated by MTT assay. Values were expressed as mean ± SD

The MTT assay showed that Gen (0–25 μM) alone had no adverse effects on PC12 cells viability, but 50 and 100 μM decreased viability compared with the control group (*p* < 0.05) (Fig. 1a). Incubation with Aβ_25–35_ significantly increased PC12 cells apoptosis in a dose-dependent manner at concentrations up to 20 μM, with no further increase at 80 μM (Fig. 1b). PC12 cells pretreated with Gen for 2 h prior to Aβ_25–35_ incubation indicated a bell-shaped effect of Gen on the viability of PC12 cells (Fig. 1c), with significant rescue at low Gen concentrations.

### Hoechst 33342 staining to detect PC12 cells apoptosis

**Fig. 2:**
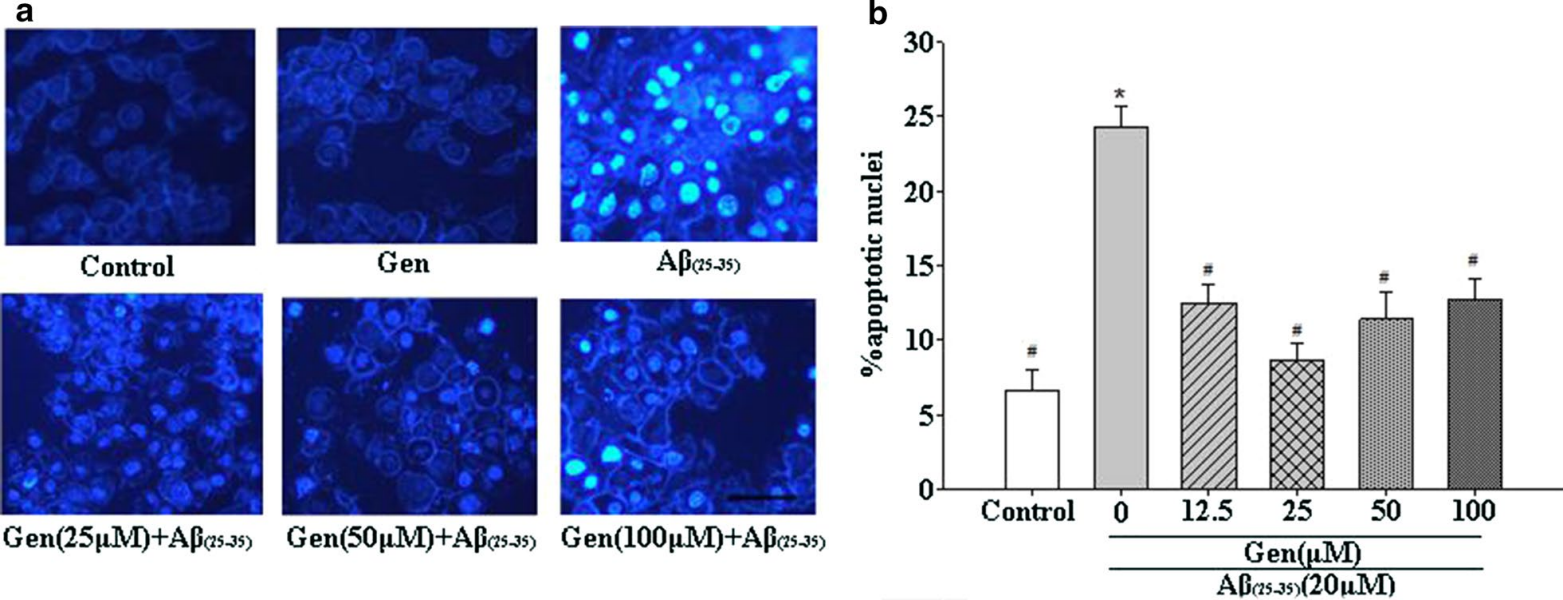
PC12 cells were stained with the DNA-binding fluorochrome Hoechst 33342. **a** Fluorescence micrographs of PC12 cells from control group, pretreated with Gen (0, 12.5, 25, 50, 100 μM) for 2 h followed by incubation with 20 μM Aβ_25–35_ for another 24 h (*scale bar* 100 μm). **b** The percentage of PC12 cells with apoptosis was estimated. *p < 0.05 compared to control; ^#^p < 0.05 compared to Aβ_25–35_ alone

Hoechst 33342 staining showed that the percentage of apoptotic cells in media containing Aβ_25–35_ was dramatically increased compared with the normal group . However, Gen pretreatment significantly decreased the apoptosis rate compared with the Aβ_25–35_ group (Fig. 2).

### Using FACS to detect PC12 cells apoptosis

**Fig. 3:**
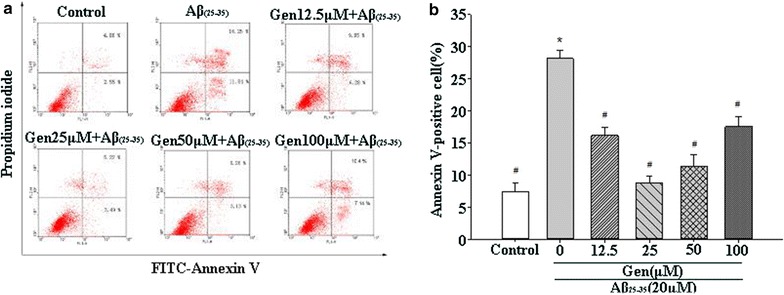
Gen pretreatment attenuation Aβ_25–35_-induced cell apoptosis. **a** Annexin-V-FITC/PI double staining of PC12 cells. **b** The *bar chart* describes the percentage distribution of apoptotic cells. Percentage of annexin-V-positive cells analysis of FACS obtained from three separate experiments and are expressed as mean ± SD, n = 3. *p < 0.05 compared to control; ^#^p < 0.05 compared to Aβ alone

The rate of cell apoptosis was measured by labeling cells with annexin-V-FITC/PI (Fig. 3a). Quantitative analysis of Annexin V-positive cells indicated that treatment cells with Aβ_25–35_ (20 μM) for 24 h significantly increased cell apoptosis, but that Gen pretreatment at 12.5–100 μM markedly decreased cell apoptosis, with the maximal protective effects seen with 25 μM Gen (Fig. 3b). Based on these results, we used 20 μM Aβ_25–35_ and 25 μM Gen in subsequent experiments.

### Gen reduced Aβ_25–35_ induced Bcl-w mRNA decreased and Bim increased

**Fig. 4:**
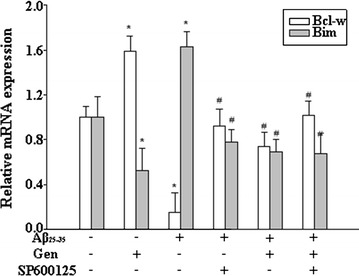
Effect of Gen on the mRNA of Bcl-w and Bim in PC12 cells detected by real-time PCR. PC12 cells were pretreated with or without Gen at concentrations of 25 μM for 2 h followed by exposure to 20 μM Aβ_25–35_ for 24 h. SP600125 (100 nM) was added to cultures 1 h prior to Aβ_25–35_. Values are expressed as mean ± SD. *p < 0.05 compared to control; ^#^p < 0.05 compared to Aβ alone

We examined the effects of Aβ_25–35_ on mRNA expression for Bcl-w and Bim, two major members of the Bcl-2 family that modulate mitochondrial apoptosis in opposing manners. Our RT-qPCR results (Fig. 4) showed that Aβ_25–35_ dramatically decreased Bcl-w and increased Bim mRNA levels, and that these changes were significantly reversed by Gen pretreatment. Furthermore, the JNK inhibitor SP600125 significantly attenuated the changes of Bcl-w and Bim mRNA expression induced by Aβ_25–35_.

### Gen attenuated release of cytochrome c and Smac induced by Aβ_25–35_

**Fig. 5:**
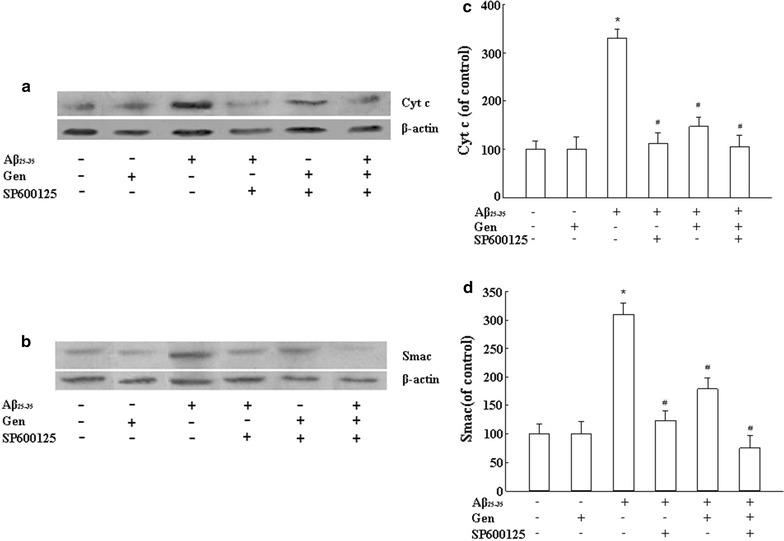
Gen reduced cytochrome c and Smac release induced by Aβ_25–35_ in PC12 cells. PC12 cells were pretreated with or without Gen at concentrations of 25 μM for 2 h followed by exposure to 20 μM Aβ_25–35_ for 24 h. SP600125 (100 nM) was added to cultures 1 h prior to Aβ_25–35_. **a** Cytochrome c levels were determined by Western blot analysis with antibody to cytochrome c. **b** Smac levels were determined by Western blot analysis with antibody to Smac. **c** Quantitated results of Cytochrome c are presented relative to control. **d** Quantitated results of Smac are presented relative to control. Densitometric analysis of Western blot obtained from three separate experiments, and data are expressed as mean ± SD, n = 3. *p < 0.05 compared to control; ^#^p < 0.05 compared to Aβ alone

Cytochrome c and Smac are released from mitochondria to the cytoplasm when mitochondrial apoptosis occurs. Western blots showed increased cytochrome c and Smac protein levels in PC12 cells incubated with Aβ_25–35_. However, pretreatment with Gen significantly attenuated this increase, as did incubation with the JNK inhibitor SP600125 (Fig. 5).

### Effect of Gen on regulation of Aβ_25–35_ induced activity of caspase-3 and JNK

**Fig. 6:**
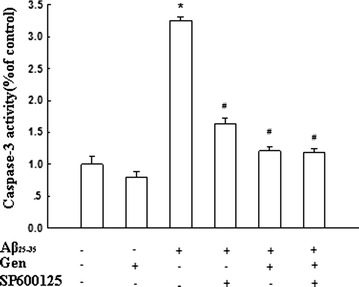
Effect of Gen on the activity of caspase-3 in Aβ_25–35_-treated PC12 cells. PC12 cells were pretreated with or without Gen at concentrations of 25 μM for 2 h followed by exposure to 20 μM Aβ_25–35_ for 24 h. SP600125 (100 nM) was added to cultures 1 h prior to Aβ_25–35_. Values were expressed as mean ± SD. *p < 0.05 compared to control; ^#^p < 0.05 compared to Aβ alone

**Fig. 7:**
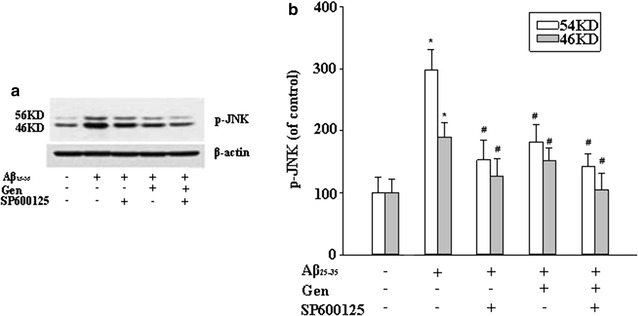
Gen attenuation Aβ_25–35_-induced JNK phosphorylation detected by Western blot. PC12 cells were pretreated with or without Gen at concentrations of 25 μM for 2 h followed by exposure to 20 μM Aβ_25–35_ for 24 h. SP600125 (100 nM) was added to cultures 1 h prior to Aβ_25–35_. **a** p-JNK levels were determined by Western blot analysis with antibody to p-JNK. **b** Quantitated results of p-JNK are presented relative to control. Densitometric analysis of Western blot obtained from three separate experiments, and data are expressed as mean ± SD, n = 3. *p < 0.05 compared to control; ^#^p < 0.05 compared to Aβ alone

Caspases are key players in the apoptotic process and play a crucial role in the execution of mitochondria-mediated apoptosis. Results (Fig. 6) showed that Gen significantly inhibited the activation of caspase-3 activity in PC12 cells induced by Aβ_25–35_. Western blot results (Fig. 7a) showed that Aβ_25–35_ significantly increased the p-JNK level in PC12 cells. However, Gen pretreatment blocked the Aβ_25–35_-induced p-JNK expression, whereas co-incubation with the JNK inhibitor SP600125 potentiated the inhibitory effect of Gen on Aβ_25–35_ induced JNK phosphorylation (Fig. 7b).

## Discussion

Aβ aggregation and formation of intracellular components of senile plaques (SP) and NFT are key steps during the pathological process of neurodegenerative disease. In vitro studies showed that the overexpression and aggregation of Aβ is an initiator of neuronal degeneration [16], and intracerebral Aβ injection provokes neuronal damage [17]. The active Aβ proteolytic fragment Aβ_25–35_ retains the capacity to induce neuronal apoptosis, although uncertainty about the relevant signaling pathways has hindered the development of specific targeted treatments; this motivated the present investigation of the interaction between the phytoestrogen Gen and Aβ toxicity.

The process of programmed cell death through activation of distinct signaling pathways, including the mitochondrial apoptotic pathway [18]—a cascade which is involved in Aβ neuronal toxicity [19]. The mitochondrial apoptotic pathway is initiated by members of the Bcl-2 family. Among these, Bcl-w is widely expressed in mammalian tissues, particularly mature brain [20]. Overexpression of Bcl-w in primary culture neurons [21] conferred protection from Aβ-induced apoptosis, thus suggesting that Bcl-w may be a constitutive inhibitor of apoptosis. In contrast, Bim is a pro-apoptotic member of Bcl-2 family, which is reported to upregulate in a variety of neuronal death paradigms [22]. Thus, silent inhibition of Bim by antisense and genetic knockout approaches can markedly decrease apoptosis of neurons [23, 24]. Later reports have shown that Aβ_25–35_ downregulates Bcl-w and upregulates Bim [21], and Gen inhibits Aβ_25–35_ induced neurotoxicity via PKC signaling pathway and regulation of the CaMKII/CREB pathway [10, 11]. Present results confirm our hypothesis that the neuroprotective agent Gen should normalize pro-apoptotic alterations in Bcl-w and Bim mRNA expression by Aβ_25–35_.

Cytochrome c—a key constituent of the electron transfer chain in cellular respiration [25]—is normally confined to the inner mitochondrial membrane. When apoptosis signaling causes irreparable mDNA damage, cells hosting these mitochondria show increased release of cytochrome c into their cytosol, suggesting a mechanistic marker for apoptosis [26, 27]. Therefore, we also monitored cytoplasmic levels cytochrome c and Smac in Aβ_25–35_-stressed PC12 cells. As expected, Aβ_25–35_ exposure increased cytosol cytochrome c and Smac, whereas pretreatment with Gen rescued PC12 cells from these increases, indicating protection of mitochondria. Moreover, activity of caspase enzymes play a major role in the modulation of apoptosis [28], and histopathology shows co-localization of hyperphosphorylated tau protein and caspases in the brainstem of AD patients [29]. In the present study we found that pretreated with Gen attenuated the caspase-3 activity induced by Aβ_25–35_. This finding implies that Gen interferes with Aβ induced apoptosis in PC12 cells through effects on the mitochondrial apoptotic pathway.

JNK activation is closely linked to distinct apoptotic stimuli, whereas silencing of JNK signaling can protect against apoptosis of neurons [30]. In addition, results of studies in vitro and in vivo show that alterations of JNK pathways are associated with pathogenesis and apoptosis of neurons in AD [31]. Importantly, evidence shows that pretreatment with the JNK inhibitor SP600125 prior to Aβ_25–35_ exposure blocked expression of Bcl-2 family members, including Bcl-w and Bim [21]. This (in conjunction with the present results) implies that Gen may influence Bcl-2 family expression through JNK signaling. Indeed, these results showed that Gen significantly reduced the phosphorylation of JNK, suggesting that amelioration by Gen of Aβ_25–35_-induced changes in the mitochondrial apoptotic pathway is mediated by JNK activation.

The pharmacological basis of Gen’s effects may be related to its estrogenic profile. Bagheria et al. [32] found that Gen treatment ameliorated the Aβ induced impairment of short-term spatial memory via an estrogenic pathway in rats. Indeed, several studies have shown that estrogen can promote the regeneration of stressed neurons, and can protect neurons from death [33]. Moreover, clinical studies show that postmenopausal women treated with estrogen replacement therapy had less memory deficits compared to women not receiving estrogen treatment [34]. An epidemiological survey also showed that estrogen replacement therapy was associated with significantly reduced risk of AD in aged women. However, estrogen therapy is a double-edged sword, imparting neuroprotective effects but also increasing the risk for neoplastic transformation in certain non-neuronal cell types [35]; this trade-off has limited the use of estrogen for protection against dementia in women.

On the other hand, recent studies showed that several phytoestrogens such as Gen, puerarin, and tanshinone have neuronal protective effects and few side effects, thus favoring further investigations into the clinical use of these compounds. Researchers have shown positive effects of Gen in cancer [36], cognitive dysfunction [10], and heart disease [37]. Gen can ameliorate Aβ-induced pathology and astrogliosis [38, 39]. Gen has a different tissue-specific agonist–antagonist profile than estrogen since, while it can be neuroprotective, it does not cause cancer in the uterus and other tissues in analogy to tamoxifen, which is an antagonist on some tissues but an agonist in others. This background motivates the present investigation into the neuroprotective attributes of Gen.

## Conclusion

Our results are consistent with the hypothesis that Gen can attenuate Aβ_25–35_ induced PC12 cells apoptotic through inhibition of Aβ_25–35_ induced JNK activation, JNK-dependent decreased of Bcl-w and increased of Bim, along with attenuation of cytochrome c and Smac release from the mitochondria, and reduced caspase-3 activity. Furthermore, findings upon concomitant treatment with JNK inhibitor SP600125 and Gen showed that additional factors may mediate resistance to Aβ_25–35_-triggered apoptosis. We conclude that Gen—a major active ingredient of soybean isoflavones—possessing a good safety profile and merits further investigation as a treatment to suppress neuronal apoptosis.

## Additional file

**Additional file 1:** The data of the results of mRNA and activity of caspase 3 and caspase 8.

## Abbreviations

Gen: genistein
p-JNK: p-c-Jun N-terminal kinase
Aβ_25–35_: GSNKGAIIGLM
AD: Alzheimer’s disease
NFT: neurofibrillary tangles
JNK: c-Jun N-terminal kinase
Smac: second mitochondrion-derived activator of caspase
PBS: phosphate buffered saline
FACS: fluorescence-activated cell sorting
PI: propidium iodide
TBS: tris-buffered saline
