# Supplementary material for: Genistein protects against Aβ25–35 induced apoptosis of PC12 cells through JNK signaling and modulation of Bcl-2 family messengers
Source: BMC Neurosci. 2017 Jan 12;18:12. doi: 10.1186/s12868-016-0329-9 (PMC5234099; doi:10.1186/s12868-016-0329-9)
Supplement: Supplementary file 1 — Additional file 1: The data of the results of mRNA and activity of caspase 3 and caspase 8. [file 12868_2016_329_MOESM1_ESM.docx]

**Additional file**

Additional file 1: Fig. 1. Effect of Gen on the mRNA and activity of caspase 3 and caspase 8 in Aβ_25-35_-induced PC12 cells. Values were expressed as mean±SD.*P＜0.05,**P＜0.01 compared to control; #P＜0.05 compared to Aβ alone.
